# Parental adverse childhood experiences, parenting styles, and stress: a comparison of child psychopathology diagnostic groups

**DOI:** 10.3389/frcha.2026.1921885

**Published:** 2026-10-23

**Authors:** K. Pfaff, S. Lion, J. Hussong, H. Honecker-Gebauer, E. Möhler

**Affiliations:** 1 Child and Adolescent Psychiatry, Saarland University, Homburg, Germany; 2 Department of Child and Adolescent Psychiatry, Psychosomatics, and Psychotherapy, Saarland University Hospital, Homburg, Germany

**Keywords:** ADHD, adverse childhood experiences, child psychopathology, functional incontinence, internalizing disorders, parental stress, parenting style

## Abstract

**Background:**

Adverse childhood experiences (ACEs) are associated with long-term impairments in physical and mental health and may influence the development of the next generation. Although parental ACEs have been linked to parenting difficulties and increased parental stress, their role across child psychiatric disorders remains unclear.

**Methods:**

This cross-sectional observational study examined associations between parental ACEs, parenting style, parental stress, and child psychopathology in a sample of 76 children and adolescents (aged 6–17 years) across three diagnostic groups: ADHD (*n* = 29), internalizing disorders (*n* = 32), and functional incontinence (*n* = 15). Parents completed the ACE-D, EEI, ESF, and CBCL 6–18R.

**Results:**

Descriptively, mothers reported higher mean ACE scores than fathers (1.5 vs. 0.9). Parental ACE scores correlated with child externalizing symptoms (*ρ* = .386, *p* < .01) and parental stress (*ρ* = .257, *p* < .05), but not with internalizing symptoms. Parenting style differed between groups, with parents of children with ADHD reporting greater strictness than parents of children with internalizing disorders. No direct association between ACE scores and parenting style was found. The overall group difference in parental stress was significant, but no *post-hoc* group comparison remained significant. Love/appreciation was negatively associated with both symptom dimensions and parental stress. Regression analysis identified externalizing symptoms (*β* = .454, *p* < .001) and role restriction (*β* = .336, *p* < .01) as significant predictors of parental stress (adjusted R^2^ = .46). As both, role restriction and parental stress are subscales of one questionnaire, this association may reflect common-method variance.

**Conclusion:**

Parental ACEs are associated with child externalizing symptoms and heightened parental stress, but showed no direct relationship with parenting style, suggesting ACEs operate via different risk factors. In the multiple regression model, parental ACE scores were no longer significantly associated with parental stress once child externalizing symptoms and role restriction were taken into account. These findings underscore the relevance of positive parenting, child externalizing symptoms, and perceived role restriction in understanding parental stress. Routine ACE assessment may identify at-risk families and inform interventions targeting parental stress and positive parent–child relationships.

## 1 Introduction

Adverse childhood experiences (ACEs) describe a range of potentially traumatic events occurring during childhood, including different forms of maltreatment (e.g., emotional, physical, and sexual abuse or neglect) as well as household dysfunction such as parental separation, domestic violence, substance abuse, or mental illness within the family (1). Over the past decades, a growing body of research has demonstrated that exposure to ACEs is associated with numerous adverse outcomes across the lifespan. Individuals with a history of ACEs show increased risks for physical health problems, including obesity and chronic diseases, as well as mental health disorders such as depression, anxiety, and attention-deficit/hyperactivity disorder (ADHD), impaired educational attainment, and socioeconomic disadvantage later in life (1, 2).

Several studies have demonstrated associations between parental ACE exposure and increased risk of child psychopathology. Children of parents with high ACE scores show higher levels of both internalizing and externalizing symptoms, including anxiety, depression, behavioral problems, and hyperactivity (3, 4). For example, Schickedanz et al. (4) found that children of parents with four or more ACEs were significantly more likely to show behavioral problems and emotional disturbances compared with children of parents without ACE exposure. Moreover, parental ACEs have been associated with developmental delays in early childhood (5) and increased risks for behavioral problems and emotional dysregulation in later childhood. One proposed mechanism involves parenting behavior: parents with a history of adversity may be more likely to show harsh or inconsistent discipline, reduced responsiveness, and less supportive parent–child interactions (6–8).

The type of child psychopathology also shapes parental stress and parenting behavior. Parents of children with ADHD report higher parenting stress than parents of children with other disorders or healthy controls (9), and meta-analytic evidence confirms that parents of children with ADHD use significantly less positive parenting and more harsh discipline (10). For internalizing disorders, such as depression and anxiety disorder, overprotective, authoritarian, and psychologically controlling parenting have been consistently identified as correlates of anxiety and depressive symptoms in children (11, 12). In contrast, the role of parenting in functional incontinence has received comparatively little research attention: a longitudinal study identified low authoritative parenting style as a predictor of primary enuresis (13), and mothers of children with constipation reported more overprotection and strictness than mothers of healthy controls (14). Maternal postpartum mental health problems have also been linked to increased risk of lower urinary tract symptoms in children (15, 16). Previous studies have reported increased parental stress in families of children with incontinence (17), but little is known about the role of parental childhood adversity and parenting behavior in these families.

Despite this growing body of research, a number of questions remain unanswered. First, many previous studies have focused primarily on maternal ACE exposure (2, 18–20), whereas paternal experiences have received less attention. Second, most studies have examined community or pediatric samples (4, 5, 18–20), while relatively few studies have investigated these associations in clinical populations of children with diagnosed psychiatric disorders. Finally, aalthough parental ACEs, parenting, and child psychopathology have been linked (21–23), and parenting stress has been studied within single diagnostic groups (9), these factors have rarely been examined together across different diagnostic groups.

Therefore, the aim of the present study was to examine the associations between parental adverse childhood experiences, parenting style, parental stress, and child psychopathology in a clinical sample of children and adolescents with three different diagnostic groups: ADHD, internalizing disorders, and functional incontinence. We further explored whether parental ACE exposure differs between these diagnostic groups and whether ACEs are associated with parental stress and parenting behavior. Based on previous research, we hypothesized that higher parental ACE scores would be associated with higher levels of parental stress, less adaptive parenting styles, and higher levels of child psychopathology.

## 2 Materials and methods

### 2.1 Study design and participants

A cross-sectional observational study was conducted at the outpatient clinic of the Department of Child and Adolescent Psychiatry, Psychosomatics, and Psychotherapy, Saarland University Hospital, Homburg, Germany. The study was approved by the local ethics committee (No. 110/21) and registered at the German Clinical Trial Registry (DRKS No.: DRKS00025986). All procedures were conducted in accordance with the Declaration of Helsinki. Written informed consent was obtained from all caregivers before participation.

Children aged 6–17 years with a recent primary ICD-10 diagnosis of ADHD, an internalizing disorder, or functional incontinence were recruited consecutively alongside their parents. Three diagnostic groups were defined:

- **Group 1—**ADHD**:** ICD-10 F90.0
- **Group 2—**Internalizing disorders: ICD-10 F32, F33, F40-F43, F93
- **Group 3—**Functional incontinence: ICD-10 F98.0, F98.1, N39.4

In group 2, patients with F43 diagnoses were only included in case of PTSD diagnosis (F43.1) or adjustment disorders (F43.2) with internalizing symptoms. Patients were assigned to diagnostic groups by their primary diagnosis at recruitment. Following informed consent, at least one parent or caregiver completed the study questionnaires. Sociodemographic data and CBCL scores were obtained from clinic files. Exclusion criteria were: age ≥18 years, insufficient German language proficiency, severe neurological comorbidity, or insufficient diagnostic clarity.

### 2.2 Instruments

Parental ACEs were assessed using the German version of the Adverse Childhood Experiences Questionnaire (ACE-D) (24). The ACE-D contains 10 questions about experienced emotional, physical or sexual abuse, neglect, and household dysfunction (e.g., parental divorce/separation or domestic violence). Each item is answered with yes (1) or no (0). The total ACE sum score reflects the number of parental ACEs. The ACE-D shows good reliability and validity (25, 26).

Parental stress was measured by the German Parental Stress Questionnaire (27). This instrument consists of 38 items in four scales, which are answered on a five-point Likert scale: Parental Stress (17 items), Role Restriction (7 items), Social Support (7 items) and Partnership (7 items). Scores are reported as stanines according to norms for school children. The questionnaire has good psychometric properties (27).

The 54 items of the German Parenting Style Inventory [Eltern-Erziehungsstil-Inventar (EEI)] assess four categories of parenting behavior (love/appreciation, rigour/strictness, independence, religiousness) and two further scales representing parental cooperation with the partner and with the school. It is answered on a four-point Likert scale. Norm data are provided as mean item responses (1–4) for all scales. Validity and reliability of the questionnaire are given (28).

Child psychopathology (externalizing, internalizing and total symptoms) was measured using the CBCL 6–18R. The CBCL consists of eight subscales and three composite scales (internalizing, externalizing, total) and is a valid and reliable instrument for measuring psychopathology in children and adolescents (29).

### 2.3 Statistical analysis

Statistical analysis was performed with IBM SPSS Statistics 23. Descriptives were described by means and standard deviations (SD) for interval variables and by absolute and relative numbers for categorical variables. Group differences for categorical variables were calculated by Chi^2^ tests and Fisher's Exact tests. *Post hoc* results for non-parametric data were calculated by *z*-tests for column proportions in the *χ*^2^ contingency table (using Bonferroni correction). Group differences for interval data were calculated by one-way ANCOVAs controlling for the covariates children's age and sex. Following a significant main effect, *post hoc* pairwise comparisons were performed using Bonferroni correction on the estimated marginal means. In case of violation of the homogeneity of variance, a robust ANCOVA using HC3 robust standard errors was conducted. Results were regarded as significant with *p* < 0.05. To examine predictor variables for parental stress, a regression analysis was calculated. Spearman rank-order correlations were used for the correlational analyses, because several variables, including the ESF and EEI scores were not normally distributed. The group comparisons and correlational analyses were exploratory. No correction for multiple comparisons was applied. Given the modest subgroup sizes, findings should therefore be interpreted with corresponding caution.

## 3 Results

### 3.1 Descriptive data

A total of 76 children (48.7% male, mean age = 11.4 years, SD = 3.4) and their parents were included: *n* = 29 with ADHD, *n* = 32 with internalizing disorders, and *n* = 15 with functional incontinence. Groups differed significantly in mean age (*p* < .001) and sex distribution (*p* < .001), as expected given the typical age and sex distributions of these disorders. Group 2 included main diagnoses of depression (*n* = 11), anxiety disorders (F40, F41, F93) (*n* = 12), OCD (*n* = 3), PTSD and adjustment disorder with emotional symptoms (F43) (*n* = 6).

The ACE-D was completed by mothers alone in *n* = 55 cases, fathers alone in *n* = 8 cases, and both parents in *n* = 13 cases (12 mothers, 1 father). The ESF and EEI were completed by mothers in *n* = 62 cases, fathers in *n* = 12 cases, and another caregiver in *n* = 1 case. In the one remaining family neither parent returned the ESF/EEI, which leads to total *n* = 75 complete ESF/EEI sets. In *n* = 2 (of these), ESF/EEI were completed by a different parent than the ACE, i.e., in *n* = 73, ACEs were completed by the same parent as the EEI/ESF. Those cases were included in correlation analyses. When both parents completed the ACE-D, the score of the parent who also completed the ESF/EEI was used in all analyses. Accordingly, the correlation analyses were based on *n* = 73 and the multiple regression on *n* = 61 families with complete data on all model variables. To assess internal consistency in the sample, Cronbachs Alpha were calculated for the ESF and EEI subscales. Regarding CBCL, only T-values of the scales were available from the data set. As the CBCL is a well-established and extensively validated instrument for assessing psychological symptoms in children and adolescents, we rely on the documented reliability and validity of the standardized instrument (29). Regarding ESF, internal consistency ranges from acceptable (*α* = .760, Social Support) to excellent (*α* = .919, Partnership). In the EEI subscales, internal consistency ranges in the acceptable to excellent spectrum (*α* = .740, Scale “Independence” to *α* = .926, Scale “Parental cooperation with partner”), except for the scale “Parental cooperation with school” (*α* = .469). Because of this low internal consistency, results involving this subscale (Tables 2, 3) should be interpreted with caution.

Descriptive statistics are presented in Table 1. The mean age as well as the gender distribution differed between the diagnosis groups, reflecting the specific manifestation characteristics known in the disorders. Accordingly, externalizing and internalizing symptoms are highest in the ADHD and internalizing groups, respectively.

**Table 1:** Descriptive data.

	**Total (*n* = 76)**	**ADHD (A) (*n* = 29)**	**Internalizing (I) (*n* = 32)**	**Functional Incontinence (F) (*n* = 15)**	***p*** ^a^
Mean age in years (SD)	11.4 (3.4)	9.6 (2.2)	13.5 (2.8)	8.2 (2.5)	<.001* (A < I)
Boys in % (n)	48.7 (37)	72.4 (21)	25.0 (8)	53.3 (8)	<.001* (A > I)
Mean parental age in years (SD)	41.2 (6.7)	40.5 (7.1)	42.6 (6.9)	39.4 (4.9)	.250
Parents separated/divorced in % (*n*)	35.5 (27)	31.0 (9)	40.6 (13)	33.3 (5)	.722
CBCL Total, mean T-Value (SD)	65.1 (10.7)	67.6 (8.6)	65.4 (11.7)	59.4 (10.9)	.060
CBCL Internalizing, mean T-Value (SD)	64.1 (10.2)	62.0 (8.0)	68.0 (11.9)	60.2 (7.3)	.030^b^* (F < I)
CBCL Externalizing, mean T-Value (SD)	60.4 (12.3)	64.8 (10.7)	57.3 (12.6)	57.6 (12.8)	.040* (A > I)

a One-way ANOVA; Chi^2^-test.

b Welch test.

A, ADHD; I, Internalizing; F, Functional Incontinence.

* *p* < .05.

CBCL, Child Behavior Checklist.

### 3.2 ACEs, parental stress, and parenting style in diagnostic groups

ACE scores, parental stress, and parenting style by diagnostic group are presented in Table 2. Adjusted mean scores (SE) are reported alongside the unadjusted means and SDs in Table 2 for the ACE, ESF and EEI scales. Mean ACE scores did not differ significantly between diagnostic groups for either mothers or fathers. Across the total sample, mothers reported higher mean ACE scores than fathers (1.5 vs. 0.9). ACE scores were numerically higher in the functional incontinence group for both mothers (M = 2.6) and fathers (M = 2.5). This difference was not statistically significant in mothers, due to small sample sizes in the subgroups, statistical testing for paternal ACEs was omitted. After adjustment for child age and sex, the overall group effect for parental stress was significant (*p* = .040), but no *post hoc* pairwise comparison remained significant. Regarding parenting style, parents of children with ADHD reported significantly higher strictness (*p* = .012; A > I) compared with parents of children with internalizing disorders. Love/appreciation did not differ significantly across groups. The distribution of specific ACE types for mothers and fathers is shown in Figures 1,2.

**Table 2:** Group differences in ACE score (mothers/fathers), parenting style, and parental stress (adjusted to age and sex).

Mean ACE scores 0–10 (SD) [adjusted mean scores (SE)]					
	**Total (*n* = 76)**	**ADHD (A) (*n* = 29)**	**Internalizing (I) (*n* = 32)**	**Functional Incontinence (F) (*n* = 15)**	***p*** ^a^
in mothers (*n* = 68)	1.5 (2.0)	1.5 (1.8) [1.5 (0.4)]	1.0 (1.6) [1.0 (0.5)]	2.6 (2.8) [2.7 (0.6)]	n.s.^b^
Min–Max	0–8	0–6	0–6	0–8	
≥4 ACEs in % (*n*)	19.1 (13/68)	17.9 (5/28)	11.5 (3/26)	35.7 (5/14)	n.s.^c^
in fathers (*n* = 21)	0.9 (1.8)	1.7 (2.7) [1.5 (0.8)]	0.3 (0.5) [0.3 (0.5)]	^_^^d^individual scores: 0, 5	^_^ ^d^
Min–Max	0–7	0–7	0–1	^_^ ^d^	
≥4 ACEs in % (*n*)	9.5 (2/21)	16.7 (1/6)	0 (0/13)	^_^^d^(1/2)	^_^ ^d^
Parental Stress Questionnaire (ESF), mean stanines 1–9 (SD) [adjusted mean stanines (SE)]					

	**(** ***n* = 75)**	**(** ***n* = 29)**	**(** ***n* = 32)**	**(** ***n* = 14)**	
Parental Stress	5.9 (2.0)	6.8 (1.5) [6.7 (0.4)]	5.4 (2.2) [5.5 (0.4)]	5.3 (2.1) [5.3 (0.6)]	.040* (*post hoc* n.s.)
Role Restriction	5.0 (1.9)	5.0 (2.0) [4.8 (0.4)]	4.9 (2.0) [5.1 (0.4)]	5.1 (2.0) [5.0 (0.6)]	.858
Social Support	4.5 (1.7)	4.5 (2.0) [4.5 (0.4)]	4.3 (1.7) [4.4 (0.4)]	4.6 (1.4) [4.4 (0.5)]	.974
Partnership (*n* = 61)	4.7 (2.0)	4.9 (1.8) [4.8 (0.4)]	4.2 (2.2) [4.4 (0.4)]	5.4 (1.7) [5.1 (0.6)]	.686
Parenting Style Inventory (EEI), mean item responses (1–4) (SD) [adjusted mean item responses (SE)]					

	**(** ***n* = 75)**	**(** ***n* = 29)**	**(** ***n* = 32)**	**(** ***n* = 14)**	
Love/Appreciation	3.6 (0.3)	3.6 (0.3) [3.6 (0.1)]	3.6 (0.4) [3.7 (0.1)]	3.7 (0.3) [3.7 (0.1)]	.825
Rigour/Strictness	2.6 (0.5)	2.8 (0.4) [2.8 (0.1)]	2.4 (0.5) [2.4 (0.1)]	2.5 (0.5) [2.5 (0.1)]	.012** (*post hoc* A > I)
Independence	3.3 (0.4)	3.4 (0.5) [3.4 (0.1)]	3.2 (0.4) [3.1 (0.1)]	3.3 (0.2) [3.4 (0.1)]	.090
Religiousness	1.8 (0.6)	1.9 (0.8) [2.0 (0.1)]	1.6 (0.5) [1.6 (0.1)]	1.8 (0.6) [1.9 (0.2)]	.738
Parental cooperation with partner	2.9 (1.0)	3.0 (1.0) [3.0 (0.2)]	2.7 (0.9) [2.8 (0.2)]	3.1 (0.9) [3.0 (0.3)]	.895
Parental cooperation with school^e^	3.1 (0.5)	3.1 (0.5) [3.1 (0.1)]	3.0 (0.5) [2.9 (0.1)]	3.2 (0.3) [3.2 (0.1)]	.279

a One-way ANCOVA.

b Due to violations of homogeneity of variance ANCOVA using HC3 robust standard errors was conducted.

ᶜ Fisher's exact test.

d For the paternal functional incontinence subgroup (*n* = 2), only the individual raw scores are reported; mean, standard deviation, percentage, model-based adjusted mean and significance testing were omitted, as estimates derived from two observations are not meaningfully interpretable.

e The “parental cooperation with school” subscale showed low internal consistency (*α* = .469); findings involving this subscale should therefore be interpreted with caution.

* *p* < .05.

** *p* < .01.

–. [ ] = adjusted mean scores and standard errors (SE). ACE, Adverse Childhood Experiences.

**Figure 1:**
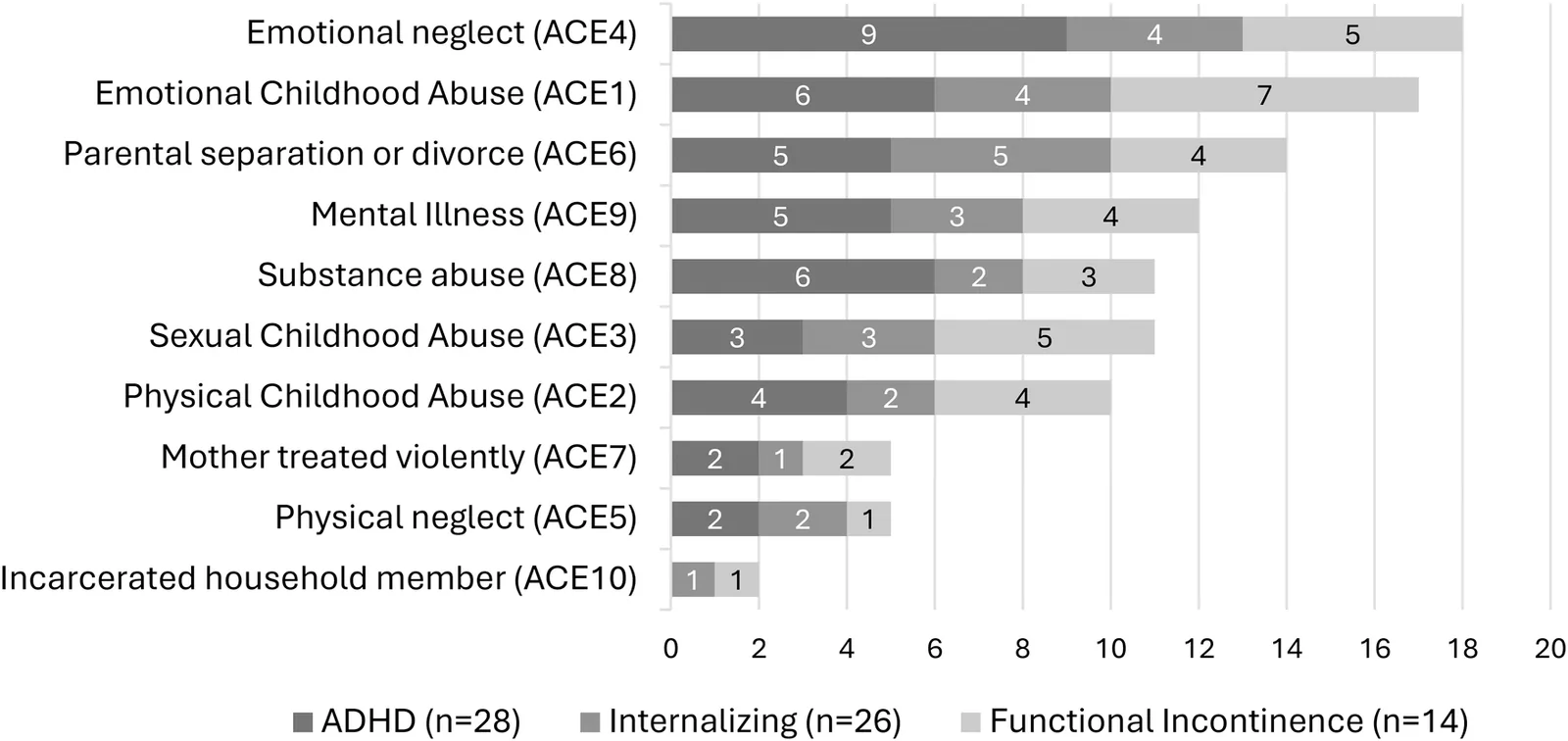
Number of specific ACEs reported by mothers (*n* = 68), by diagnostic group. ADHD, Attention-Deficit/Hyperactivity Disorder.

**Figure 2:**
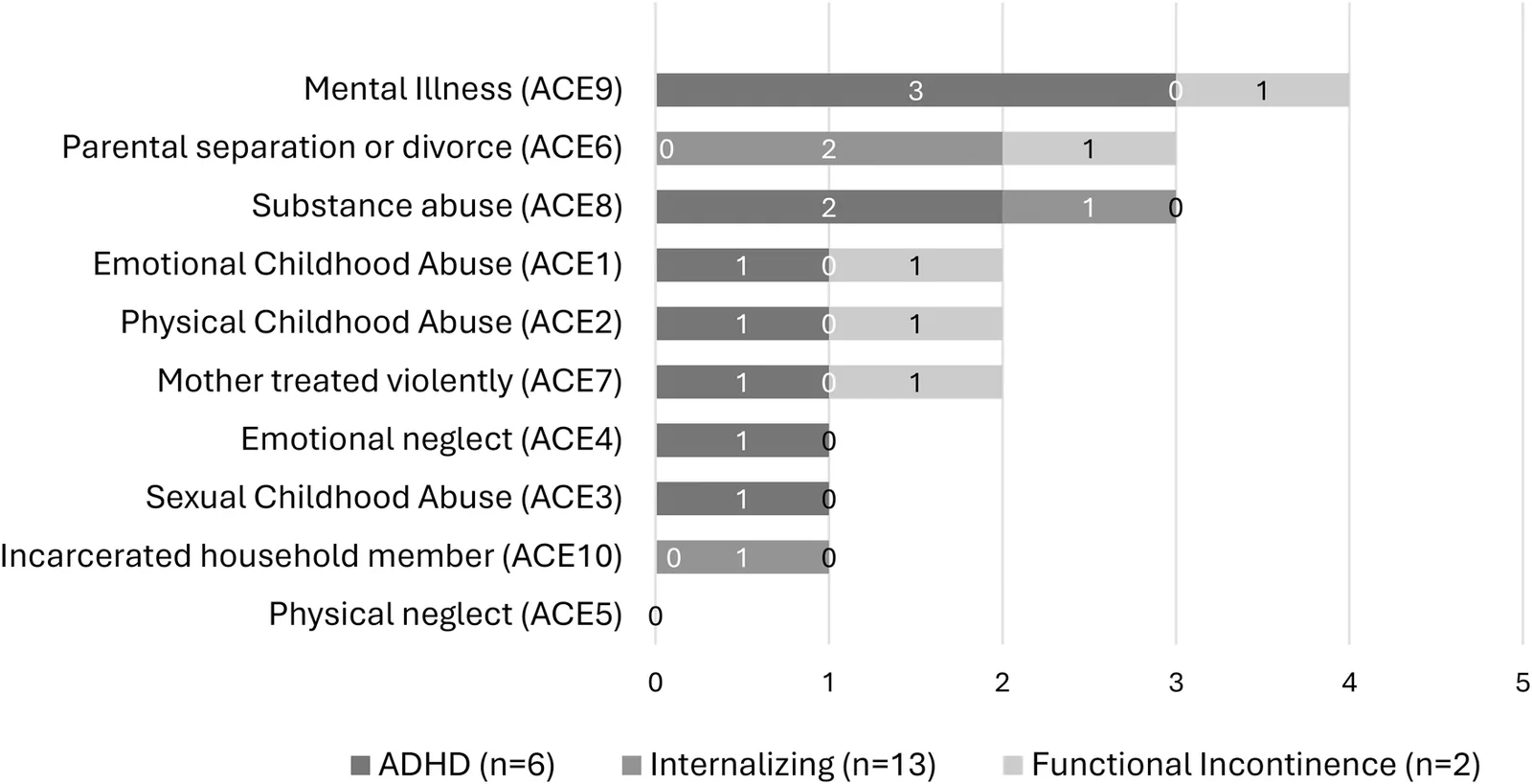
Number of specific ACEs reported by fathers (*n* = 21), by diagnostic group. ADHD, Attention-Deficit/Hyperactivity Disorder.

Among mothers, the most common ACEs were emotional neglect, emotional abuse, and parental separation. Among fathers, mental illness in the household, parental separation, and substance abuse were most frequent.

### 3.3 Associations among ACE score, psychopathology, parental stress and parenting style

Correlational analyses were calculated to explore associations between ACEs, psychopathology, parental stress and parenting style. Because the variables deviated from normal distribution (Shapiro–Wilk test: *p* < .001) and some variables showed elevated kurtosis values, Spearman rank-order correlations were used to examine associations between the study variables.

In *n* = 3 cases, the ACE was completed by a different parent than the ESF and EEI. These subjects were excluded from correlation analysis. The multiple regression model was based on the 61 families with complete data on the outcome and all included predictors; cases with missing data on any model variable were excluded. Results are presented in Table 3. The ACE sum score was significantly associated with child externalizing symptoms (*ρ* = .386, *p* < .01) and parental stress (*ρ* = .257, *p* < .05), but not with internalizing symptoms. Parental stress correlated positively with externalizing symptoms (*ρ* = .570, *p* < .01) and role restriction (*ρ* = .431, *p* < .01), and negatively with partnership (*ρ* = –.395, *p* < .01). Higher parental love/appreciation was negatively associated with internalizing symptoms (*ρ* = –.255, *p* < .05), externalizing symptoms (*ρ* = –.360, *p* < .01), and parental stress (*ρ* = –.341, *p* < .01). Internalizing symptoms correlated negatively with parental cooperation with partner (*ρ* = –.306, *p* < .05), while externalizing symptoms correlated negatively with partnership (*ρ* = –.302, *p* < .05). No significant associations were found between ACE scores and any EEI parenting dimension (all *p* > .28).

**Table 3:** Spearman correlations of parental ACE score, child internalizing and externalizing symptoms, parental stress and parenting style.

	**ACE score**	**Internalizing symptoms (CBCL INT T-value)**	**Externalizing symptoms (CBCL EXT T-value)**	**Parental Stress (ESF Stanine)**
CBCL				
Internalizing symptoms	–		.462**	–
Externalizing symptoms	.386**	.462**		.570**
Parental Stress Questionnaire (ESF)				
Parental Stress	.257*	–	.570**	
Role Restriction	–	–	–	.431**
Social Support	–	–	–	–
Partnership	–	–	–.302*	–.395**
Parenting Style Inventory (EEI)				
Love/Appreciation	–	–.255*	–.360**	–.341**
Rigour/Strictness	–	–	–	–
Independence	–	–	–	–
Religiousness	–	–	–	–
Parental cooperation with partner	–	–.306*	–	–
Parental cooperation with school^a^	–	–	–	–

Only significant correlations are shown; non-significant cells are marked with –; ACE, Adverse Childhood Experiences; CBCL, Child Behavior Checklist; ESF, Elternstressfragebogen; EEI, Eltern-Erziehungsstil-Inventar.

* *p* < .05.

** *p* < .01.

a The “parental cooperation with school” subscale showed low internal consistency (*α* = .469); findings involving this subscale should therefore be interpreted with caution.

To examine predictor variables for parental stress, a regression analysis was calculated. As the ESF scale “partnership” is only calculated when the parent is in a current relationship. This was valid for *n* = 61, only those were included in the regression analysis. The multiple regression analysis for parental stress was significant [F(5, 55) = 11.3, *p* < .001; adjusted R^2^ = .46]. Child externalizing symptoms (*β* = .454, *p* < .001) and role restriction (*β* = .336, *p* < .01) were the only significant independent predictors. ACE sum score, partnership, and love/appreciation did not reach significance in the full model. Regression coefficients (Betas) are presented in Table 4.

**Table 4:** Regression coefficients and significant predictors in the regression analysis of parental stress.

Predictor variables	Beta (SE)	Standardized Beta
Constant	2.263 (3.02)	
ACE sum score	.045 (.097)	.046
CBCL Externalizing score	.072 (.017)	.454***
Role Restriction	.136 (.040)	.336**
Partnership	–.075 (.040)	–.187
Love/Appreciation	–.020 (.092)	–.022

ACE, Adverse Childhood Experiences; CBCL, Child Behavior Checklist; SE, Standard Error.

** *p* < .01.

*** *p* < .001.

A comparison of maternal ACE prevalences with normative data from the representative German population sample of Witt et al. (30) reveals a substantially elevated burden in the present clinical sample, particularly for relational and attachment-relevant ACE categories, as shown in Table 5. While prevalences for parental separation and substance-related household problems were broadly comparable to population reference values, emotional neglect and emotional abuse were reported by approximately twice as many mothers in the present sample as in the general population. Most notably, the rate of childhood sexual abuse was markedly elevated (16.2% vs. 4.3%), indicating a selective overrepresentation of severe traumatic experiences in this clinical population. Physical abuse and growing up in a household with mental illness were also reported more frequently than in the reference sample.

**Table 5:** Prevalence of individual ACE categories in mothers and fathers of the present clinical sample compared with German population norms (30).

ACE category (ACE-D)	Mothers (*N* = 68) *n* (%)	Fathers (*N* = 21) *n* (%)	Witt et al. (30) population sample *n* (%)
Emotional neglect (ACE4)	18 (26.5%)	1 (4.8%)	13.4%
Emotional abuse (ACE1)	17 (25.0%)	2 (9.5%)	12.5%
Parental separation (ACE6)	14 (20.6%)	3 (14.3%)	19.4%
Mental illness in household (ACE9)	12 (17.6%)	4 (19.0%)	10.6%
Substance problems in household (ACE8)	11 (16.2%)	3 (14.3%)	16.7%
Sexual abuse (ACE3)	**11** **(16.2%)**	1 (4.8%)	**4** **.** **3%**
Physical abuse (ACE2)	10 (14.7%)	2 (9.5%)	9.1%
Domestic violence (ACE7)	5 (7.4%)	2 (9.5%)	9.8%
Physical neglect (ACE5)	5 (7.4%)	0 (0%)	4.3%
Incarceration of household member (ACE10)	2 (2.9%)	1 (4.8%)	3.5%

ACE-D, German version of the Adverse Childhood Experiences Questionnaire. Bold values indicate rates notably exceeding German population norms.

## 4 Discussion

The present study investigated associations between parental ACEs, parenting style, parental stress, and child psychopathology in a clinical sample of children and adolescents with three different diagnoses. Parental stress showed an overall group difference, but no specific group comparisons were significant in *post hoc* analyses. ACE scores correlated with child externalizing symptoms and parental stress, but not with internalizing symptoms or any parenting style dimensions. Regression analyses identified child externalizing symptoms and parental role restriction as the primary predictors of parental stress, while ACE score did not independently predict stress after controlling for other variables.

### 4.1 Prevalence of parental ACEs

The prevalence of parental ACEs in this clinical sample is consistent with findings from other pediatric and clinical populations. Shah et al. (31) found that 64% of parents in a general hospital medicine service reported ≥1 ACE and 19% had ≥4 ACEs. The descriptively higher mean ACE scores among mothers in the present sample likely reflect established gender differences in vulnerability to and reporting of relational adversity, particularly emotional abuse and neglect (4). Yet, this difference was not formally tested and, because 13 couples contributed data for both parents, the maternal and paternal samples are not fully independent; the comparison should therefore be read as descriptive. The functional incontinence group showed numerically higher ACE scores for mothers, though this did not reach statistical significance. This may partly reflect a selection effect: since we only included children aged 6 and older, older children with persistent incontinence, who may come from more burdened families, could be overrepresented. The corresponding paternal values rest only on two fathers and are therefore not interpreted.

As detailed in the Results and shown in Table 5, maternal ACE prevalences in the present clinical sample exceeded the German population values reported by Witt et al. (30), most clearly for the relational and attachment-relevant categories, as emotional neglect, emotional abuse and sexual abuse, as well as for physical abuse and household mental illness.

These findings are consistent with models of intergenerational transmission that identify relational and attachment-relevant forms of adversity, particularly emotional abuse, neglect, and sexual abuse, as central risk factors for long-term impairments in stress regulation, emotional availability, and parenting behavior (21, 22). Corresponding ACE categories have been associated in the literature with lasting alterations in caregiving sensitivity and parent-child interaction quality. Against this background, it is plausible that maternal ACEs in the present study were specifically associated with elevated child psychopathology. The findings suggest that not only the cumulative number of ACEs, but particularly their relational quality and proximity to early attachment relationships, may be relevant for the intergenerational transmission. In contrast to the maternal data, paternal ACE profiles in the present sample did not show a systematic overrepresentation compared with the population reference values of Witt et al. (30). Prevalences for most ACE categories were within or below the normative range. An exception was growing up in a household with mental illness, which was reported more frequently by fathers in the present sample. Substance-related household problems and emotional and physical abuse also occurred at clinically relevant frequencies, while emotional neglect and sexual abuse were notably rare among fathers.

Although paternal ACE rates were lower overall, the reported adversities, including physical and emotional abuse and household mental illness or substance problems, represent potentially relevant risk experiences that may affect paternal stress regulation, impulse control, and emotional availability (22, 32). These experiences may manifest specifically in paternal parenting behavior through reduced availability, heightened stress reactivity, or less sensitive interaction patterns. Given that fathers are increasingly recognized as an important factor in children's emotional security and self-regulation, the consideration of their childhood adversity is also interpretively meaningful. However, due to the small number of fathers in the present sample (*n* = 21), statistically robust conclusions cannot be drawn. Associations regarding parental ACEs and child outcomes were observed primarily for the parent who completed the questionnaires, who was in most cases the mother. Whether this reflects a specific role of the primary caregiver or is attributable to the predominance of maternal reports cannot be determined from the present cross-sectional data. Associations involving externalizing behavior or parental control may differ by parental role and gender, and future studies should include both parents systematically to examine this question.

### 4.2 Parental ACEs and child psychopathology

Consistent with the literature, parental ACEs were significantly associated with child externalizing symptoms but not internalizing symptoms at the whole-sample level. This aligns with findings showing that cumulative parental adversity is linked to conduct problems, hyperactivity, and behavioral dysregulation in offspring (4, 7, 8). The lack of an association with internalizing symptoms may indicate that this pathway is more complex and depends more heavily on genetic, biological, or other environmental factors (18). Maternal ACE exposure has consistently shown a stronger relationship with child behavior problems than paternal ACEs (4), a finding attributed to *in utero* maternal effects and the greater role of mothers as primary caregivers. The relationship between ACEs and internalizing outcomes has also been linked specifically to parental depression and maternal attachment avoidance as mediators (18, 23), none of which were assessed in the present study, representing an important limitation.

Research has further shown a graded dose-response relationship between maternal ACE scores and child behavioral problems, including conduct problems, learning difficulties, and hyperactivity, partly mediated by maternal parenting styles of rejection (19). Wattanacharia et al. (33) showed, that ACEs were directly associated with the use of corporal punishment (*β* = 0.15). ACE exposure has also been identified as a significant risk factor for ADHD diagnosis and symptom severity in children (34), which helps explain why ACE scores tended to be higher among parents of children with ADHD in our sample.

### 4.3 Parenting style and child outcomes

Group differences in parenting style were observed in the strictness scale, with parents of children with ADHD reporting higher scores compared with parents of children with internalizing disorders. The most straightforward explanation for this pattern is that parents are responding to the demands of raising a child with ADHD: impulsivity, hyperactivity, and non-compliance may prompt parents to enforce firmer boundaries and structure (35). Age differences between groups may also contribute, as ADHD children were younger on average and parental strictness tends to be higher for younger children. This interpretation is consistent with evidence that parenting in ADHD is not uniformly negative: in a case-control study, parents of children with ADHD reported higher positive-parenting scores, with differences in inconsistent discipline that varied across ADHD presentations (36).

Parents of children with internalizing disorders reported lower strictness. This pattern may reflect a cautious, protective adjustment to a perceived vulnerability in the child, consistent with theoretical models linking internalizing symptoms to parental avoidance of confrontation, reduced structure, and heightened sensitivity to child distress (37, 38). Notably, love/appreciation scores did not differ across groups, suggesting that the emotional quality of the parent–child relationship is largely preserved regardless of the child's diagnosis.

No direct association was found between parental ACE scores and any EEI parenting dimension. This is consistent with the hypothesis that ACEs function as distal rather than proximal risk factors, with their effects on parenting behavior mediated by current psychological state, stress regulation capacity, and social support (39, 40). It is also possible that the EEI, which measures broad and fairly stable parenting attitudes, cannot pick up the more moment-to-moment, stress-driven changes in parenting that tend to be affected by early adversity.

### 4.4 Parental stress

Parental stress was numerically highest in the ADHD group, consistent with prior meta-analytic evidence, and particularly pronounced for the combined ADHD subtype (9). In the present sample, no *post hoc* group comparison remained significant after adjustment for child age and sex. In the regression analysis, child externalizing symptoms and parental role restriction were the only significant predictors of parental stress, together accounting for 46% of the variance. This finding highlights the central role of challenging child behavior as a correlate of parental stress and shows that feeling personally restricted by the parenting role adds meaningfully to stress, on top of the child's behavior. Because Role Restriction and Parental Stress are subscales of the same instrument (ESF; 27), their association may partly reflect common-method variance rather than a fully independent predictibe relationship. Although the two subscales comprise distinct, non-overlapping items (Parental Stress, 17 Items; Role Restriction, 7 Items), shared method and response-style variance between subcales of one questionnaire cannot be ruled out. This coefficient should therefore be interpreted with caution, and future work should test the model with exogenous predictors only. Another mechanism linking parental ACEs to child outcomes is parental stress. Parents who experienced adversity in their own childhood may have fewer psychological resources to cope with the challenges of parenting, especially when raising children with behavioral or emotional problems. Higher parental ACE scores have been associated with higher levels of parental stress and psychological distress (20), and elevated parental stress is in turn associated with more negative parenting and poorer parent-child relationships (35).

A similar pattern was observed in a previous study of children with autism spectrum disorder, where externalizing symptoms and role restriction were also identified as significant stress predictors (41), and in our own earlier investigations of children with functional incontinence, where child-related stressors were the dominant predictor (42).

The ACE sum score correlated with parental stress bivariately but did not remain significant in the regression model after accounting for child externalizing symptoms and role restriction. This attenuation is compatible with an indirect pathway (22). The finding that better co-parental partnership was linked to lower stress fits with previous research in families of children with autism (41) and with the broader idea that social support helps parents cope. Higher parental love/appreciation was also negatively associated with stress, suggesting that interventions promoting positive parent–child interactions may simultaneously reduce parental burden and improve child outcomes.

### 4.5 Clinical implications

The present findings have several implications for clinical practice in child and adolescent psychiatry. First, the results support routine screening of parental ACEs in the clinical evaluation of children presenting with psychiatric disorders, particularly for those with externalizing symptoms. Such screening creates opportunities for early trauma-focused intervention for parents at elevated risk (43, 44). Second, the finding that parental stress, rather than ACE history, is the strongest proximal predictor of family functioning suggests that reducing parental stress and role restriction should be a primary therapeutic target. Parenting programs and family-based therapies that address both child symptom management and parental emotional resources are likely to be most effective (45, 46). Third, the consistent negative associations between parental love/appreciation and both child symptom dimensions suggest that fostering emotional warmth is a meaningful protective intervention target across diagnostic groups.

### 4.6 Limitations

Several limitations should be considered. First, the sample was relatively small, particularly the functional incontinence subgroup (*n* = 15), limiting statistical power and generalizability. Second, the cross-sectional design precludes causal inference. Third, parental psychopathology was not assessed; given its established role as a mediator of intergenerational ACE effects (3, 18, 23), its omission limits interpretation of the observed associations. Fourth, retrospective self-report of ACEs is susceptible to recall bias and social desirability. Fifth, the EEI captures global parenting orientations and may lack sensitivity for situation-specific or symptom-reactive parenting changes. Sixth, the absence of a non-clinical control group limits comparison with families without child psychopathology. Seventh, the diagnostic groups differed substantially in child age and sex. Eighth, the maternal and paternal ACE data are partly non-independent, as some couples contributed both, and the paternal subgroup analyses rest on very small numbers. Further, regarding the predictor variables of parental stress, the reliance on a single assessment instrument introduces a potential common-method bias that limits the generalizability of this specific finding. Although the two subscales comprise distinct, non-overlapping items, common-method variance between subscales of the same questionnaire cannot be excluded. Regarding the correlations, transforming the data into standardized and rank-based metrics was statistically necessary, but this approach may have led to a minor loss of variance information. Consequently, the direct comparability of the correlation magnitudes across the different instruments should be interpreted with caution.

### 4.7 Strengths

Key strengths include the simultaneous assessment of parental ACEs, parenting style, parental stress, and child psychopathology within a single, consistently characterized clinical sample; the use of standardized and validated instruments; the inclusion of three clinically distinct diagnostic groups within the same study; and the testing of pre-specified hypotheses. Comparing three diagnostic groups within the same study allowed a descriptive comparison of parental ACE burden, parenting style, and parental stress across clinical groups.

## 5 Conclusion

Parental ACEs are associated with child externalizing symptomatology and heightened parental stress in clinical child psychiatric populations. In the regression model, parental ACE scores were no longer significantly associated with parental stress after child externalizing symptoms and role restriction were accounted for. This attenuation is consistent with an indirect pathway but was not tested in formal mediation analysis. No direct association between ACEs and parenting style was found, suggesting ACEs function as distal rather than proximal risk factors. The pattern of results points to a model in which early adversity functions as a background risk that becomes clinically relevant primarily through its interaction with current child behavior and parental role strain. Parents who carry a history of ACEs and are simultaneously confronted with a child showing externalizing symptoms appear to be at particular risk for elevated stress, which may in turn affect the quality of the parent-child relationship over time. Within this framework, parental warmth and appreciation was consistently associated with lower symptom levels and lower parental stress across all three diagnostic groups, underlining the clinical importance of supporting positive parent-child relationships regardless of diagnosis.

These findings support the routine assessment of parental childhood adversity in child and adolescent psychiatric practice and the implementation of family-based interventions that target parental stress reduction and the promotion of warm, responsive parenting. Interventions that specifically address parental emotional resources, including stress regulation, co-parental support, and sensitivity, are likely to benefit not only the parent but also the child's behavioral and emotional development. However, the present study has several limitations that should be kept in mind when interpreting these findings, including the relatively small sample size, the cross-sectional design, and the absence of a control group. These factors limit the strength of the conclusions that can be drawn.

Future research should use larger samples, include parental psychopathology as a mediator, and use longitudinal designs to better understand how parental ACEs are passed on across generations. Studies that combine observational measures of parenting with self-report data, and that assess both maternal and paternal ACEs systematically, would be particularly valuable.

## Data Availability

The original contributions presented in the study are included in the article/Supplementary Material, further inquiries can be directed to the corresponding author.
