# Supplementary material for: Parental adverse childhood experiences, parenting styles, and stress: a comparison of child psychopathology diagnostic groups
Source: Front Child Adolesc Psychiatry. 2026 Oct 23;5:1921885. doi: 10.3389/frcha.2026.1921885 (PMC13739703; doi:10.3389/frcha.2026.1921885)
Supplement: Supplementary file 1 [file Datasheet1.pdf]

Appendix I. Adjusted mean scores and standard errors (SE) for ACE scores, parental stress, and parenting style.

	ADHD (n = 29)	Internalizing (n = 32)	Functional Incontinence (n = 15)
	ACE scores, adjusted means (SE)		
Mothers	1.49 (0.40)	0.97 (0.48)	2.73 (0.59)
Fathers	1.52 (0.77)	0.26 (0.51)	3.25 (1.45)
	Parental Stress Questionnaire (ESF), adjusted means (SE)		
Parental Stress	6.73 (0.40)	5.47 (0.43)	5.33 (0.57)
Role Restriction	4.78 (0.39)	5.09 (0.42)	5.02 (0.56)
Social Support	4.52 (0.36)	4.44 (0.38)	4.39 (0.51)
	Parenting Style Inventory (EEI), adjusted means (SE)		
Love/Appreciation	3.62 (0.07)	3.68 (0.08)	3.67 (0.10)
Rigour/Strictness	2.85 (0.10)	2.44 (0.10)	2.52 (0.14)
Independence	3.43 (0.09)	3.11 (0.09)	3.38 (0.13)
Religiousness	1.99 (0.13)	1.59 (0.14)	1.86 (0.20)
Cooperation with partner	2.96 (0.19)	2.83 (0.21)	3.00 (0.29)
Cooperation with schools	3.15 (0.09)	2.94 (0.10)	3.21 (0.13)

Note. Values are adjusted mean scores (estimated marginal means) with standard errors (SE). All models were adjusted for child age and child sex; ACE = Adverse Childhood Experiences.
